# Annotations

**Published:** 1906-04-07

**Authors:** 


### ANNOTATIONS.

### Our Whisky Investigation.

In the leading article contained in our issue of March 3 we drew attention to the forthcoming appearance of this report. The recent prosecutions, and the consequent mass of contradictory and trade-interested statements as to what whisky should or should not be which have been showered upon medical men, would alone render advisable the report of an independent inquiry into the subject. Whisky has, however, a sterling importance to the medical profession quite apart from the vagaries of ephemeral trade polemics, and this inquiry was begun, and, indeed, finished, by us previously to the celebrated whisky case. It will be found to be exhaustive, and contains ample material for the formation of a sober judgment by impartial minds. It is exhaustive in the sense that we have taken infinite pains in selecting the samples of Irish and Scotch whisky in common consumption in the United Kingdom and America, which include whiskies on the home market, home whiskies exported and sold in the United States, and the home or rye whiskies of America. This investigation cannot fail to be of general interest, as an inquiry into the injuriousness to health of immature whisky, and the relative therapeutic values of different kinds of whisky is a field of research likely to yield results important both to the hygienist and to the practical physician.

### Dr. Doyen's Cancer Cure.

Yet another cancer cure has come and gone. Dr. Doyen's serum, which, like so many of its fellow-specifics, has revelled in the delighted welcome of the lay press in this country, has failed to justify itself in the hands of dispassionate and unbiassed observers in our own medical ranks. Dr. Alexander Paine and Dr. David Morgan have, at the request of the authorities of the Cancer Hospital, conducted a series of trials of the efficacy of the serum and have pronounced against it. The serum in question is obtained by the immunisation of horses against an organism which Dr. Doyen found in many tumours both simple and malignant, and which he named in consequence the *Micrococcus neoformans*. He reported that he was able, by inoculation of this organism into animals, to produce new growths, and regarded all growths as the result of an infection comparable with that of tuberculosis and actinomycosis. This statement Drs. Paine and Morgan have failed to corroborate. The organism described was found by these observers in 11 out of 44 tumours examined; but they found a streptococcus in 13 cases, and are satisfied that micro-organisms are frequently present in new growths, without apparently possessing any causal relationship to the growths in question. Moreover, the serum itself does not possess the first essential of therapeutics—it is not harmless. On the contrary, it seems certain that in many cases injections of the serum lead to grave constitutional disturbance. The pity of the whole business is the easy publicity accorded to comparatively untried schemes of treatment. One might think that common humanity would dictate to editors of lay papers a wiser reserve: idly to overset the hardly-bought resignation of the victims of cancer is an unworthy practice, and one well calculated to multiply the already sufficient distresses of these unfortunates.

### Nerve Grafting.

Any form of treatment that gives hope of relieving, even in a small degree, the loss of power of activity which results from such a disease as infantile paralysis deserves serious attention. Dr. Spiller and two of his colleagues publish in the March number of "The American Journal of the Medical Sciences" a paper upon nerve-grafting, and refer to cases of limited paralysis in which considerable increase of muscular power and variety of movement have followed this form of surgical treatment. The paper, however, deals chiefly with that distressing form of movement, athetosis, which at first sight would not appear to be likely to receive benefit from dealing with nerves. It is needless to say that the origin of the evil is central, not peripheral; but in the case reported the movements in the flexor muscles were much more violent than in the extensors—a fact which seems to have suggested the idea that some relief might be afforded by an operation upon the nerves. Those nerves mainly supplying the flexor muscles were therefore divided and joined to those supplying the extensor muscles. The median and ulnar were joined by lateral anastomosis to the musculo-spiral nerve, but the musculo-cutaneous and the circumflex nerves were completely divided, and the peripheral end of one joined to the central end of the other, and *vice versa*. As a result it is needless to say that the arms were much weakened, but at the end of two and a half months sufficient power had returned to enable him to follow his occupation of selling papers, and he is said to have been "very happy over his improved condition." Apparently the only involuntary movements which remained were in muscles of the right shoulder supplied by nerves which had not been operated upon. Dr. Spiller remarks that it might be thought probable that since the irregular movements are of central origin, the prevention of their manifestation by interfering with the nerve-supply to the muscles might eventually result in the appearance of movements outside the distribution of the nerves operated upon. At the time of writing, however, seven and a half months after the operation, no such return had taken place, and Dr. Spiller hoped that, so far as the movements were concerned, the man was permanently relieved. He adds that no one who had seen the man before and after the operation could doubt "that he was far more comfortable than at any previous period in his life."

